# Effect of supplemental methionine on health and performance of receiving beef heifers

**DOI:** 10.1093/tas/txac113

**Published:** 2022-08-21

**Authors:** Madeline S Grant, Hannah F Speer, N Daniel Luchini, Dale A Blasi, Evan C Titgemeyer

**Affiliations:** Department of Animal Sciences and Industry, Kansas State University, Manhattan, KS 66506, USA; Department of Animal Sciences and Industry, Kansas State University, Manhattan, KS 66506, USA; Adisseo USA Inc., Alpharetta, GA 30022, USA; Department of Animal Sciences and Industry, Kansas State University, Manhattan, KS 66506, USA; Department of Animal Sciences and Industry, Kansas State University, Manhattan, KS 66506, USA

**Keywords:** haptoglobin, methionine, receiving cattle

## Abstract

Methionine supplementation can improve immune function in transition dairy cattle. Our objective was to determine if supplemental methionine could improve health and performance of newly received growing cattle. Crossbred heifers (*n* = 384; 222 kg initial body weight; southeastern U.S. origin) were received in four truckloads (blocks) over 9 d. Heifers were weighed at arrival. The following day (d 0) cattle were vaccinated for viral and clostridial diseases, received 2.5 mg tulathromycin/kg body weight, and were stratified within the blocks by arrival body weight to 1 of 8 pens containing 12 heifers each. Within blocks, pens were assigned to 1 of 2 treatments: 0 (control) or 0.1725% Smartamine M to provide 0.1035% metabolizable methionine to the diet. Cattle were limit-fed at 2.2% of body weight daily (dry matter basis) on a diet containing 40% wet corn gluten feed, 34.5% dry-rolled corn, 10% corn silage, 7.5% supplement, 4% alfalfa hay, and 4% prairie hay. Pen weights were measured weekly to determine the feed offered the following week. Individual body weight and tail-vein blood samples were collected on d 0, 14, and 45. Plasma haptoglobin was measured to assess acute-phase protein response. Incidences of morbidity (1.6% for control, 2.6% for Smartamine M) and mortality (0.5% for both control and Smartamine M) were low. Between d 0 and 45, no differences were observed for average daily gain (1.24 vs. 1.27 kg/d; control vs. Smartamine M, *P* = 0.55) or gain:feed (0.107 vs. 0.110, *P* = 0.28), although dry matter intake was 1.3% greater (*P* < 0.01) for control than Smartamine M due to differences in diet dry matter concentration. An interaction between treatment and linear effect of day was detected for plasma haptoglobin (*P* < 0.05); over time, haptoglobin increased more for control (2.15, 2.28, and 2.95 mg/mL at 0, 14, and 45 d) than for Smartamine M (2.35, 2.37, and 2.58 mg/mL). Supplemental methionine may alleviate acute-phase protein responses in stressed receiving cattle.

## INTRODUCTION

Methionine is an essential amino acid that is often first limiting for growth in cattle when microbial protein is the protein is the primary source of metabolizable protein (Richardson and Hatfield, 1978). This is a result of the low methionine content of microbial protein synthesized in the rumen (Orskov, 1982). In addition to its quantitatively most important role in protein synthesis, when methionine is converted to *S*-adenosylmethionine, it serves as the most widely used methyl group donor in the body (Finkelstein, 1990). As *S*-adenosylmethionine, methionine participates in over a hundred essential methylation reactions throughout the body (Lobley, 1992), including synthesis of creatine and phosphatidylcholine as well as methylation of histones, deoxyribonucleic acid, and ribonucleic acid (Walker, 1979). Phosphatidylcholine is the predominant phospholipid in lipoproteins (Skipski et al., 1967), and is essential for hepatic export of triglycerides in very light density lipoproteins (Cole et al., 2012). Methionine also serves as a key precursor to intracellular antioxidants glutathione and taurine (Brosnan and Brosnan, 2006). Dysregulated lipid transport and antioxidant imbalance can both contribute to inflammation in the body (Drackley, 1999; Sordillo et al., 2009), so adequate methionine supply may help control inflammation in the body.

Because amino acids are rapidly degraded by ruminal microbes, ruminally protected amino acids must be fed to ensure that they are available for absorption in the small intestine. One of the most widely studied and utilized forms of ruminally protected methionine is Smartamine M (Adisseo USA Inc., Alpharetta, GA), which utilizes a pH-sensitive co-polymer coating to resist ruminal degradation but allows solubilization in the abomasum and absorption in the small intestine.

In the transition period, dairy cattle face numerous physiological stressors due to reduced dry matter intake near calving and increased nutrient partitioning toward fetal growth and the onset of lactation (Drackley, 1999). Supplementation of ruminally protected methionine to transition dairy cattle has been shown to improve performance, health, and immune function (Ardalan et al., 2010; Osorio et al., 2014; Zhou et al., 2016a, 2016b). These improvements are likely a result of corrected antioxidant balance and/or reduced inflammation in the peripartal period (McFadden et al., 2020). Newly received beef cattle face stress associated with marketing and transport to the feedlot including commingling, pathogen exposure, and low dry matter intake. As a result, high-risk receiving cattle often become ill shortly after feedlot arrival. Because these stressors may be physiologically similar to those of dairy cattle in the peripartal period, methionine may have potential as an immunomodulator in receiving cattle. To our knowledge, no previous work evaluating the effects of supplementing ruminally protected methionine on receiving cattle health and inflammation has been conducted. Our objective was to evaluate the effect of supplemental methionine on performance, health, and acute-phase protein response in high-risk receiving beef cattle.

## MATERIALS AND METHODS

### Animals and Experimental Diets

A total of 384 crossbred heifers (222 ± 4.9 kg initial body weight) of southeastern U.S. origin were purchased from auction markets in Tennessee, transported and commingled at an order buyer’s facility in Dixon, TN, then transported 1,086 km to the Kansas State University Beef Stocker unit where they were received over 9 d from October 4 to October 13, 2018. Cattle were blocked by truckload (4) and stratified by individual arrival body weight within a block to 8 pens containing 12 animals each. Within block, pens were assigned randomly to 1 of 2 treatments creating 16 pens/treatment, with a total of 32 pens. All pens (9.1 × 15.2 m) were soil surfaced and had a concrete fenceline bunk (9.1 m) and 3.6-m apron. Experimental diets were formulated to contain 1.32 Mcal NEg/kg dry matter. Experimental diets (Table 1) were offered at 2.2% of body weight daily (dry matter basis) and contained either 0 (control) or 0.1725% Smartamine M. Because Smartamine M was included as a percentage of the diet, Smartamine M intake (i.e., metabolizable methionine intake) increased as dry matter intake increased throughout the trial. During the first 14 d of the experiment (i.e., during step up) heifers supplemented with Smartamine M received on average 7.5 g/d Smartamine M (i.e., 4.5 g/d supplemental metabolizable methionine). For entire 45 d trial, heifers supplemented with Smartamine M received on average 9 g/d Smartamine M (i.e., 5.4 g/d metabolizable methionine). Smartamine M was mixed with dry-rolled corn before being added to the diet. The mixture was prepared in a paddle mixer; corn and Smartamine M were combined and mixed for 60 s to ensure an even distribution of Smartamine M without damaging the pH-sensitive coating on the product. Two bins were utilized to store corn, one with dry-rolled corn (control) and the other containing the dry-rolled corn-Smartamine M mixture.

**Table 1.** Composition of diets fed to receiving heifers

	Treatment	
Item	Control	Smartamine M
Ingredient, % of dry matter		
Corn, dry rolled	34.5	—
Smartamine M-corn mixture^1^	—	34.5
Wet corn gluten feed^2^	40.0	40.0
Corn silage	10.0	10.0
Alfalfa hay	4.0	4.0
Prairie hay, chopped	4.0	4.0
Supplement^3^	7.5	7.5
Nutrient composition, % of dry matter		
Dry matter, % as is	62.8	62.6
Organic matter	93.5	93.2
Neutral detergent fiber	22.1	23.7
Acid detergent fiber	8.7	9.6
Starch	53.8	54.3
Crude protein	13.2	13.3

Smartamine M and dry-rolled corn were combined and mixed for 60 s in a paddle mixer according to Smartamine M user guide instructions. The mixture contained 99.5% dry-rolled corn and 0.5% Smartamine M. The Smartamine M diet contained 0.1725% Smartamine M.

Sweet Bran, Cargill Animal Nutrition, Blair, NE.

Supplement pellets formulated to contain (dry matter basis) 10.6% crude protein, 8.7% Ca, 0.62% P, 4.6% NaCl, 0.70% K, 0.20% Mg, 5.1% fat, and 330 mg/kg monensin (Rumensin; Elanco, Greenfield, IN). Supplement ingredients were (as % of dry matter) 70.7% wheat middlings, 23.4% CaCO_3_, 5.0% NaCl, 0.35% soybean oil, 0.18% Rumensin 90, 0.11% ZnSO_4_, 0.08% MnSO_4_ (32%), 0.06% vitamin E (500,000 IU/kg), 0.05% CuSO_4_, 0.01% Se (0.99%), 0.007% ethylenediamine dihydroiodide (50 grain), 0.004% vitamin A (650,000 IU/g).

Upon arrival, heifers were individually weighed and received an individual identification ear tag. An ear notch was collected from each animal and immediately analyzed for persistent infection with bovine viral diarrhea virus using a rapid visual enzyme-linked immunosorbent assay (IDEXX BVDV PI X2 Test, IDEXX Laboratories, Inc., Westbrook, ME); one heifer was identified as positive for persistent infection and was not used in the experiment. Animals not displaying illness were assigned randomly to pens containing 12 heifers and offered 0.5% body weight (dry matter basis) prairie hay and ad libitum access to water overnight.

The following morning (d 0), heifers were individually weighed, assigned an ear tag for pen number, and were vaccinated for respiratory and clostridial disease. For respiratory pathogens, Pyramid 5 + Presponse SQ (Boehringer Ingelheim Vetmedica Inc., St. Joseph, MO), a modified-live vaccine against infectious bovine rhinotracheitis virus, bovine viral diarrhea virus types I and II, parainfluenza_3_ virus, and bovine respiratory syncytial virus, was administered. For clostridial pathogens, Vision 7 Somnus with Spur (Merck Animal Health, Madison, NJ) was used. Animals were treated on d 0 for subclinical respiratory disease with 2.5 mg/kg tulathromycin (Draxxin; Zoetis, Parisippany, NJ). Heifers were also treated on d 0 for internal parasites with 10% fenbendazole (Safe-Guard; Merck Animal Health, Madison, NJ) and external parasites with pour on ivermectin (Bimectin, Bimeda US, Oakbrook Terrace, IL). On d 14, all heifers were revaccinated for respiratory pathogens with Vista 5 SQ (Merck Animal Health, Kenilworth, NJ), a modified-live vaccine for infectious bovine rhinotracheitis virus, bovine viral diarrhea virus I and II, parainfluenza_3_ virus, and bovine respiratory syncytial virus.

Individual body weights were measured on d 0, at revaccination (d 14), and at the conclusion of the study (d 45). Pen weights were measured weekly (d 14, 21, 28, 35, and 45) using a pen scale (Rice Lake Weighing Systems, Rice Lake, WI). Weekly pen weights were used to calculate the feed offered for the following week. Animals were fed once daily at 0700 h using a Roto-Mix feed wagon (Roto-Mix, Dodge City, KS). The two experimental diets were prepared identically except dry rolled corn [control] was replaced with the Smartamine M-corn mixture (Table 1). When transitioning from the Smartamine M diet to control, the feed wagon was emptied first by the auger and then manually where all reachable remaining feed was removed and discarded. Approximately 100 kg of corn silage was added to the wagon as a flushing agent, mixed for 60 s, and removed via wagon auger and manual cleaning to prevent treatment carryover. Cattle were transitioned to the treatment diets by offering 1% of body weight (dry matter basis) on d 0 and increasing feed offered by 0.2% of body weight each day if the previous day’s feed was totally consumed until the pen reached a dry matter intake of 2.2% body weight daily. During step-up, refusals were left in the bunk; if greater than approximately 5% refusals were present, feed call was decreased accordingly to avoid excessive refusals the following day. By d 14 all pens were fully stepped up to feed delivered at 2.2% body weight (dry matter basis) and consistently ate all feed offered prior to the next day’s feeding.

Heifers were observed twice daily for clinical signs of illness including depression, anorexia, gauntness, and ocular or nasal discharge. Animals showing signs of morbidity were walked to treatment facilities where rectal temperature was measured, and clinical illness score was determined as described by Hanzlicek et al. (2016). Clinical illness scores were defined as 1) normal and healthy, 2) slightly ill, with mild depression or gauntness, 3) moderately ill, with severe depression/labored breathing/ocular or nasal discharge, or 4) severely ill, near death with little response to human approach. Animals with a rectal temperature ≥ 40 °C and a clinical illness score ≥ 2 were treated. Upon the first morbidity, animals were treated with florfenicol and flunixin meglumine (300 mg/mL and 16.5 mg/mL, respectively; Resflor, Merck Animal Health, Madison, NJ). If symptoms did not improve within 72 h of the first treatment, the second morbidity was declared and animals were treated with enrofloxacin (100 mg/mL; Baytril 100, Bayer Animal Health, Shawnee Mission, KS). Upon the third morbidity, heifers were treated with oxytetracycline (200 mg/mL; Bio-Mycin 200, Boehringer Ingelheim Vetmedica, Inc., St. Joeseph, MO). At the third treatment, animals were considered chronic and removed from the trial.

### Sample Collection and Analysis

Feed ingredient and total mixed ration samples were collected weekly and frozen at −20 °C until analysis. Previously frozen feed and ingredient samples were composited on a biweekly basis, dried in a forced-air oven at 55 °C for 48 h, and ground through a 1-mm screen using a Thomas Wiley Mill (Thomas Scientific, Swedesboro, NJ). All samples were analyzed in duplicate. Ground samples were dried for 24 h at 105 °C in a forced-air oven for total dry matter determination and then heated to 450 °C in a muffle furnace for 18 h to measure organic matter content. Samples were analyzed for neutral detergent fiber using alpha-amylase and sequentially analyzed for acid detergent fiber using an ANKOM Fiber Analyzer (Model 200, ANKOM Technology, Macedon, NY). Starch content was measured by the method of Herrera-Saldana and Huber (1989) with glucose measured using a commercial colorimetric assay (Autokit Glucose, FUJIFILM Wako Diagnostics U.S.A. Corp., Mountain View, CA). Nitrogen content of samples was analyzed using a LECO TruMac N Analyzer (LECO Corporation, Saint Joseph, MI). Measured N content was multiplied by 6.25 to determine the crude protein content of the samples.

Prior to feeding on d 0, 14, and 45 a coccygeal vein blood sample was collected from each animal into a 10-mL evacuated tube (BD Vacutainer; Beckton, Dickinson, and Company, Franklin Lakes, NJ) containing sodium heparin. Following collection, tubes were immediately inverted several times and stored on ice until centrifuged at 1,200 × g at 4 °C for 20 min. Plasma was harvested and stored in 2-mL microcentrifuge tubes at −20 °C until analysis for haptoglobin concentration to assess inflammation and acute-phase protein response. Plasma haptoglobin concentrations were measured by colorimetric assay based on peroxidase activity following the methods of Cooke and Arthington (2013).

### Calculations

Average pen body weight was calculated for d 0 based on individual weights collected at processing. All subsequent average pen body weights (d 14, 21, 28, 35, and 45) were based on pen scale weights. Day 0, 14, and 45 weights were used to calculate average daily gain and gain:feed. Throughout the trial, only two pens experienced death loss; all calculations for performance data were made with data from dead heifers removed.

### Statistical Analyses

Performance data were analyzed as a randomized block design using the mixed procedure of SAS (version 9.4; SAS Institute Inc., Cary, NC) with a model including the fixed effect of treatment and the random effect of the block.

A large proportion of plasma samples collected for haptoglobin measurement contained hemolysis to some degree, which interfered with the haptoglobin assay. Thus, samples containing any visible hemolysis were not analyzed for haptoglobin concentration. As a result, only 695 individual observations of plasma haptoglobin concentration were available across the three blood sampling days. To generate pen means for plasma haptoglobin concentration for each of the three sampling days, individual observations were analyzed using the mixed procedure of SAS with a model including fixed effects of pen, day, and pen × day as well as the random effect of heifer(pen). The 96-pen means were then analyzed using the mixed procedure of SAS with fixed effects in the model including treatment, day, and treatment × day as fixed effects and block as a random effect. Day was considered a repeated measure with spatial power as the covariance structure. Treatment by day interactions was evaluated using contrasts for assessing treatment × time interactions, with linear and quadratic effects constructed for unequal spacing of sampling days. Significance was declared at *P* ≤ 0.05 and tendencies at 0.05 ≤ *P* ≤ 0.10.

## RESULTS

### Animal Performance

Performance data are presented in Table 2. Dry matter intake was greater d 0 to 14 and d 0 to 45 for control than for Smartamine M (*P* ≤ 0.01) but did not differ between treatments over d 14 to 45 (*P* = 0.54). This was the result of differences in dietary dry matter among treatments as analyzed from weekly feed samples. Because cattle were fed based on predicted ingredient and dietary dry matter, these differences were not measured until after the trial’s conclusion. No differences in body weight (*P* > 0.65), average daily gain (*P* > 0.52), or gain:feed (*P* > 0.28) were observed at any time point throughout the trial.

**Table 2.** Effect of Smartamine M on performance of beef heifers

	Treatment		SEM	*P*-value
Item	Control	Smartamine M		
No. of pens	16	16		
No. of animals	191	191		
Body weight, kg				
d 0	222	222	4.9	0.70
d 14	242	242	2.9	0.87
d 21	251	251	2.5	0.88
d 28	259	259	2.4	0.66
d 35	267	268	2.9	0.87
d 45	278	279	3.0	0.65
Average daily gain, kg/d				
d 0 to 14	1.44	1.44	0.167	0.95
d 14 to 45	1.15	1.19	0.049	0.52
d 0 to 45	1.24	1.27	0.064	0.55
Dry matter intake, kg/d				
d 0 to 14	4.43	4.34	0.044	0.01
d 14 to 45	5.65	5.63	0.051	0.54
d 0 to 45	5.28	5.21	0.045	<0.01
Gain:feed, kg/kg				
d 0 to 14	0.148	0.152	0.0187	0.70
d 14 to 45	0.093	0.096	0.0034	0.45
d 0 to 45	0.107	0.110	0.0064	0.28

### Health and Acute-Phase Protein Response

Very low incidence of morbidity and mortality were observed for this trial (Table 3); morbidity and mortality data were not analyzed statistically. A total of 8 animals were treated once (2.08%) for respiratory illness, 5 from the Smartamine M treatment (2.60%) and 3 from the control treatment (1.56%). Two previously treated animals died during the trial, one from each treatment, resulting in mortality rates of 0.52% for each treatment. One of the deceased animals (Smartamine M treatment) was considered chronically infected with respiratory illness and removed from the trial prior to death. The other (control treatment) died within 24 h of the second treatment for respiratory illness.

**Table 3.** Effects of Smartamine M supplementation on morbidity and mortality in beef heifers

	Treatment	
Item	Control	Smartamine M
Morbidity, %		
Treated once	1.56	2.60
Treated twice	0.52	0.52
Treated thrice^1^	0.00	0.52
Mortality, %	0.52	0.52

Heifers requiring three treatments were considered chronic and removed from the experiment.

**Figure 1.**
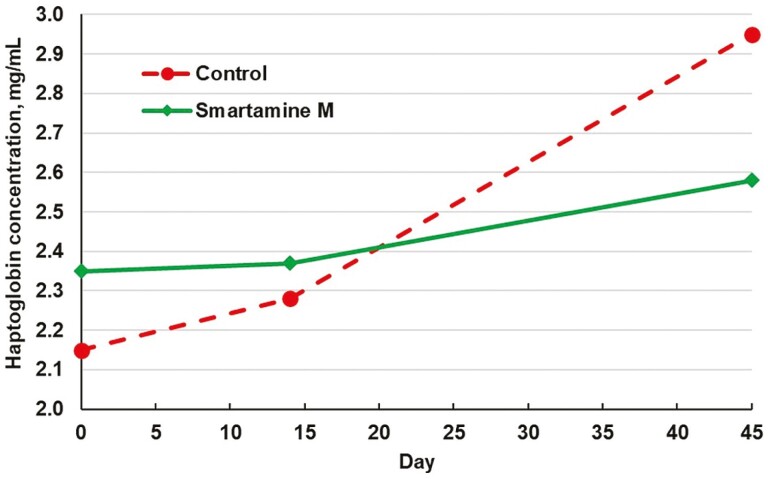
Effect of Smartamine M (Adisseo USA Inc., Alpharetta, GA) on plasma haptoglobin concentrations in receiving beef heifers. Treatment × linear day interaction, *P* = 0.05, SEM = 0.22.

For plasma haptoglobin, an interaction between dietary treatment and linear effect of day was observed (*P* = 0.05; Figure 1). Over the duration of the trial, plasma haptoglobin concentrations for the Smartamine M treatment group remained relatively stable. Control cattle had numerically lower d-0 haptoglobin than did cattle receiving Smartamine M, but control cattle then demonstrated increases over the duration of the trial. It appears that supplemental methionine mitigated this increase in plasma haptoglobin over time.

## DISCUSSION

### Animal Performance

In our study, heifers were limit-fed at a targeted 2.2% of body weight daily (dry matter basis). In the early phase of the trial (i.e., d 0 to 14), dry matter intake was greater for control heifers than for heifers supplemented with Smartamine M; however, this resulted from small differences in dry matter concentration of feed samples, and, although statistically significant, the differences were not considered important.

Work investigating the effects of supplementing ruminally protected methionine on growth and finishing performance in beef cattle has shown variable effects. In 280-kg steers fed corn- and corn silage-based diets, Deetz et al. (1985) observed no differences in average daily gain but lower dry matter intake in steers fed two levels of ruminally protected methionine compared to unsupplemented controls. Feed efficiency was greater for steers supplemented with ruminally protected methionine. Wright and Loerch (1988) observed no differences in dry matter intake, average daily gain, or feed efficiency when ruminally protected methionine was supplemented to finishing steers fed high-moisture corn-based diets. Limited response to methionine supplementation in the cattle of our trial, Deetz et al. (1985), or Wright and Loerch (1988) may be explained by either a lack of methionine deficiency or possibly another amino acid limiting performance increases. Titgemeyer et al. (1988) concluded that cattle-fed corn-based diets are most likely first limited by lysine rather than methionine. Therefore, in our cattle, which were fed a corn- and corn-byproduct-based diet and did not experience large amounts of clinical disease, it is not particularly surprising that we did not observe improvements in growth performance with supplementation of ruminally protected methionine.

Improved lactational performance (i.e., increased milk yield, milk fat, and protein yield) in postpartum dairy cows supplemented with ruminally protected methionine in the peripartum period has been reported (Osorio et al., 2013; Zhou et al., 2016b; Batistel et al., 2017). This may in part be a result of a corrected methionine deficiency; however, postpartum dry matter intake was also greater in cows supplemented with ruminally protected methionine in these studies. Improved dry matter intake following calving may explain increased lactational performance in the cows of Osorio et al. (2013), Zhou et al. (2016b), and Batistel et al. (2017). Our heifers were limit-fed, so improvements in average daily gain resulting from improved voluntary dry matter intake were not possible.

### Health and Acute-Phase Protein Response

Despite being subjected to numerous stressors prior to arrival (e.g., marketing, commingling, transport, pathogen exposure, feed restriction, etc.), our cattle had an unexpectedly low incidence of respiratory illness. It appears that our heifers’ immune function was not significantly challenged and, as a result, any effect of supplementation with ruminally protected methionine on general health could not be detected. One explanation for our low morbidity incidence is the use of metaphylaxis at initial processing, which was used to limit the expression of any respiratory illness existing at arrival (before the methionine treatment could be effective). In a similar study conducted at this research facility with metaphylaxis, Spore et al. (2019) observed an average 12% incidence of first-treatment respiratory disease in heifers of similar size and origin. Step et al. (2008) observed 31.9% incidence of respiratory illness in commingled auction-market cattle that did not receive antibiotic treatment upon arrival, markedly greater than that of Spore et al. (2019) or our cattle. Typically, metaphylaxis is expected to reduce first-treatment respiratory illness rates by approximately 50% (Wileman et al., 2009). Because our cattle were relatively healthy throughout the trial’s duration, we are unable to draw conclusions about the ability of ruminally protected methionine to reduce clinical illness in receiving cattle.

Our heifers maintained relatively stable concentrations of plasma haptoglobin between d 0 and 14 regardless of treatment; however, by d 45 control heifers had greater haptoglobin concentrations than heifers fed Smartamine M. Haptoglobin is a positive acute-phase protein produced by the liver during an inflammatory response (Ackerman, 2017); for this reason, it serves as useful a marker of general inflammatory status in the body. Our results indicate that the increase in haptoglobin (i.e., inflammation) observed in control cattle by d 45 was mitigated by supplementation of ruminally protected methionine. It is possible that plasma haptoglobin concentrations may have spiked and returned to baseline sometime between d 0 and 14 (i.e., likely due to transportation and/or initial processing stress); however, it is not certain that treatment would have had adequate time to quantitatively affect acute-phase protein response within the first week of the experiment. In transition dairy cows, variable haptoglobin responses to supplementation with ruminally protected methionine have been observed; in some cases, ruminally protected methionine decreased plasma haptoglobin (Zhou et al., 2016a; Batistel et al., 2018) whereas in others ruminally protected methionine had no effect on haptoglobin (Osorio et al., 2014; Vailati-Ribioni et al., 2017). In our cattle, because ruminally protected methionine mitigated inflammation without altering performance, it is likely that this response was due to methionine’s role in metabolism rather than as an amino acid limiting protein deposition. It is not clear why haptoglobin concentrations increased over time in control cattle that were clinically healthy in appearance.

### Conclusion

Our data suggest that methionine was not likely first limiting for growth performance in our heifers. Because our cattle were predominantly healthy, we were unable to characterize any effects of methionine supplementation on clinical illness. In contrast, our observation that methionine supplementation reduced haptoglobin late in the trial suggests that it mitigated inflammation at the physiological level. Further work evaluating the effects of methionine and other methyl group sources on health and inflammation in high-risk cattle would be helpful to determine if ruminally protected methionine has utility as an immunomodulator for receiving cattle.

## Notes

Contribution no. 22-282-J from the Kansas Agricultural Experiment Station.

## References

[CIT0001] Ackermann, M. R. 2017. Inflammation and healing. In: Zachary, J. F. editor. Pathologic basis of veterinary disease. 6th ed. St. Louis (MO): Elsevier; p. 73–131.

[CIT0002] Ardalan, M., K. Rezayazdi, and M. Dehghan-Banadaky. 2010. Effect of rumen-protected choline and methionine on physiological and metabolic disorders and reproductive indices of dairy cows. J. Anim. Physiol. Anim. Nutr. 94:e259–e265. doi:10.1111/j.1439-0396.2009.00966.x. 20455967

[CIT0003] Batistel, F., J. M. Arroyo, A. Bellingeri, B. Saremi, C. Parys, E. Trevisi, F. Cardoso, and J. J. Loor. 2017. Ethyl-cellulose rumen-protected methionine enhances performance during the periparturient period and early lactation in Holstein cows. J. Dairy Sci. 100:7455–7467. doi:10.3168/jds.2017-12689. 28711252

[CIT0004] Batistel, F., J. M. Arroyo, C. I. M. Gares, E. Trevesi, C. Parys, M. A. Ballou, F. C. Cardoso, and J. J. Loor. 2018. Ethyl-cellulose rumen-protected methionine alleviates inflammation and oxidative stress and improves neutrophil function during the periparturient period and early lactation in Holstein dairy cows. J. Dairy Sci. 101:480–490. doi:10.3168/jds.2017-13185. 29103714

[CIT0005] Brosnan, J. T., and M. E. Brosnan. 2006. The sulfur-containing amino acids: an overview. J. Nutr. 136:1636S–1640S. doi:10.1093/jn/136.6.1636S. 16702333

[CIT0006] Cole, L. K., J. E. Vance, and D. E. Vance. 2012. Phosphatidylcholine biosynthesis and lipoprotein metabolism. Biochim. Biophys. Acta. 1821:754–761. doi:10.1016/j.bbalip.2011.09.009. 21979151

[CIT0007] Cooke, R. F., and J. D. Arthington. 2013. Concentrations of haptoglobin in bovine plasma determined by ELISA or a colorimetric method based on peroxidase activity. J. Anim. Physiol. Anim. Nutr. 97:531–536. doi:10.1111/j.1439-0396.2012.01298.x. 22487219

[CIT0008] Deetz, L. E., A. M. Papas, and C. H. Benton. 1985. Performance of finishing steers fed rumen-protected methionine and/or lysine. J. Anim. Sci. 61(Suppl. 1):486 (Abstr).

[CIT0009] Drackley, J. K. 1999. ADSA Foundation Scholar Award. Biology of dairy cows during the transition period: the final frontier? J. Dairy Sci. 82:2259–2273. doi:10.3168/jds.s0022-0302(99)75474-3. 10575597

[CIT0010] Finkelstein, J. D. 1990. Methionine metabolism in mammals. J. Nutr. Biochem. 1:228–237. doi:10.1016/0955-2863(90)90070-2. 15539209

[CIT0011] Hanzlicek, G. A., D. A. Blasi, B. E. Oleen, and G. A. Anderson. 2016. A randomized field study comparing differences in core body temperature, health, and performance in crossbred beef heifers administered 2 antimicrobial products given upon arrival at a stocker facility. Prof. Anim. Sci. 32:438–444. doi:10.15232/pas.2015-01486.

[CIT0012] Herrera-Saldana, R. and J. T. Huber. 1989. Influence of varying protein and starch degradabilities on performance of lactating cows. J. Dairy Sci. 72:1477–1483. doi:10.3168/jds.S0022-0302(89)79257-2257-2. 2760309

[CIT0013] Lobley, G. E. 1992. Control of the metabolic fate of amino acids in ruminants: a review. J. Anim. Sci. 70:3264–3275. doi:10.2527/1992.70103264x. 1429303

[CIT0014] McFadden, J. W., C. L. Girard, S. Tao, Z. Zhou, J. K. Bernard, M. Duplessis, and H. M. White. 2020. Symposium review: one-carbon metabolism and methyl donor nutrition in the dairy cow. J. Dairy Sci. 103:5668–5683. doi:10.3168/jds.2019-17319. 32278559

[CIT0015] Osorio, J. S., P. Ji, J. K. Drackley, D. Luchini, and J. J. Loor. 2013. Supplemental Smartamine M or MetaSmart during the transition period benefits postpartal cow performance and blood neutrophil function. J. Dairy Sci. 96:6248–6263. doi:10.3168/jds.2012-5790. 23910549

[CIT0016] Osorio, J. S., E. Trevisi, P. Ji, J. K. Drackley, D. Luchini, G. Bertoni, and J. J. Loor. 2014. Biomarkers of inflammation, metabolism, and oxidative stress in blood, liver, and milk reveal a better immunometabolic status in peripartal cows supplemented with Smartamine M or MetaSmart. J. Dairy Sci. 97:7437–7450. doi:10.3168/jds.2013-7679. 25282419

[CIT0017] Ørskov, E. R. 1982. Protein nutrition in ruminants. London (UK): Academic Press.

[CIT0018] Richardson, C. R., and E. E. Hatfield. 1978. The limiting amino acids in growing cattle. J. Anim. Sci. 46:740–745. doi:10.2527/jas1978.463740x. 659344

[CIT0019] Skipski, V. P., M. Barclay, R. K. Barklay, V. A. Fetzer, J. J. Good, and F. M. Archibald. 1967. Lipid composition of human serum lipoproteins. Biochem. J. 104:340–352. doi:10.1042/bj1040340. 6048776 PMC1270593

[CIT0020] Sordillo, L. M., and S. L. Aitken. 2009. Impact of oxidative stress on the health and immune function of dairy cattle. Vet. Immunol. Immunopathol. 128:104–109. doi:10.1016/j.vetimm.2008.10.305. 19027173

[CIT0021] Spore, T. J., S. P. Montgomery, E. C. Titgemeyer, G. A. Hanzlicek, C. I. Vahl, T. G. Nagaraja, K. T. Cavalli, W. R. Hollenbeck, R. A. Wahl, and D. A. Blasi. 2019. Effects of high-energy programmed feeding protocol on nutrient digestibility, health, and performance of newly received growing beef cattle. Appl. Anim. Sci. 35:397–407. doi:10.15232/aas.2019-01853.

[CIT0022] Step, D. L., C. R. Krehbiel, H. A. DePra, J. J. Cranston, R. W. Fulton, J. G. Kirkpatrick, D. R. Gill, M. E. Payton, M. A. Montelongo, and A. W. Confer. 2008. Effects of commingling beef calves from different sources and weaning protocols during a forty-two-day receiving period on performance and bovine respiratory disease. J. Anim. Sci. 86:3146–3158. doi:10.2527/jas.2008-0883. 18567723

[CIT0023] Titgemeyer, E. C., N. R. Merchen, L. L. Berger, and L. E. Deetz. 1988. Estimation of lysine and methionine requirements of growing steers fed corn silage-based or corn-based diets. J. Dairy Sci. 71:421–434. doi:10.3168/jds.S0022-0302(88)79572-7. 3132487

[CIT0024] Vailati-Riboni, M., J. S. Osorio, E. Trevisi, D. Luchini, and J. J. Loor. 2017. Supplemental Smartamine M in higher energy diets during the prepartal period improves hepatic biomarkers of health and oxidative status in Holstein cows. J. Anim. Sci. Biotechnol. 8:17. doi:10.1186/s40104-017-0147-7. 28191311 PMC5295218

[CIT0025] Walker, J. B. 1979. Creatine: biosynthesis, regulation, and function. Adv. Enzymol. Relat. Areas Mol. Biol. 50:177–242. doi:10.1002/9780470122952.ch4. 386719

[CIT0026] Wileman, B. W., D. U. Thomson, C. D. Reinhardt, and D. G. Renter. 2009. Analysis of modern technologies commonly used in beef cattle production: conventional beef production versus nonconventional production using meta-analysis. J. Anim. Sci. 87:3418–3426. doi:10.2527/jas.2009-1778. 19617517

[CIT0027] Wright, M. D., and S. C. Loerch. 1988. Effects of rumen-protect amino acids on ruminant nitrogen balance, plasma amino acid concentrations and performance. J. Anim. Sci. 66:2014–2027. doi:10.2527/jas1988.6682014x. 3209509

[CIT0028] Zhou, Z., O. Bulgari, M. Vailati-Riboni, E. Trevisi, M. A. Ballou, F. C. Cardoso, D. N. Luchini, and J. J. Loor. 2016a. Rumen-protected methionine compared with rumen-protected choline improves immunometabolic status in dairy cows during the peripartal period. J. Dairy Sci. 99:8956–8969. doi:10.3168/jds.2016-10986. 27592438

[CIT0029] Zhou, Z., M. Vailati-Riboni, E. Trevisi, J. K. Drackley, D. N. Luchini, and J. J. Loor. 2016b. Better postpartal performance in dairy cows supplemented with rumen-protected methionine compared with choline during the peripartal period. J. Dairy Sci. 99:8716–8732. doi:10.3168/jds.2015-10525. 27638261

